# Direct conversion of human fibroblast to hepatocytes using a single inducible polycistronic vector

**DOI:** 10.1186/s13287-019-1416-5

**Published:** 2019-11-04

**Authors:** Maria Ballester, Miguel Bolonio, Ramon Santamaria, José V. Castell, Carmen Ribes-Koninckx, Roque Bort

**Affiliations:** 1 Experimental Hepatology Research Unit, Instituto de Investigación Sanitaria La Fe, Hospital Universitari i Politècnic La Fe and CIBERehd, 46026 Valencia, Spain; 2 Coeliac Disease and Inmunopathology Research Unit, Instituto de Investigación Sanitaria La Fe. Pediatric Gastroenterology, Hospital Universitari i Politècnic La Fe, 46026 Valencia, Spain; 3 Biochemistry and Molecular Biology Department, Universidad de Valencia, Valencia, Spain

**Keywords:** Reprogramming, Induced hepatocyte-like cells, iHEP, Polycistronic vectors, Doxycycline, Inducible

## Abstract

**Background:**

Human fibroblasts can be reprogrammed into induced hepatocyte-like cells through the expression of a set of transcription factors. Although the generation of induced hepatocyte-like cells by HNF4A, HNF1A, and FOXA3 expression has proven to be a robust experimental strategy, using multiple lentivirus results in a highly variable heterogeneous population.

**Methods:**

We designed and implemented a novel approach based on the delivery of reprogramming factors and green fluorescent protein in a single doxycycline-inducible lentiviral vector using 2A self-cleaving peptides.

**Results:**

Fibroblasts infected with the lentiviral vector can be amplified in basic fibroblast culture media in the absence of doxycycline without induction of hepatic genes. Upon switching to hepatic maturation media containing doxycycline, cells stop proliferating, activate hepatic gene transcription, and perform metabolic functions characteristic of hepatocytes.

**Conclusion:**

Our strategy can generate an unlimited source of homogeneously induced hepatocyte-like cells from different genetic background donors, capable of performing typical hepatic functions suitable for drug research and other in vitro applications.

## Background

Generation of hepatocyte-like cells from other somatic cell types to be applied for cell therapy or pharmaceutical screening is a long-standing promise. Cell therapy purposes face important challenges such as scalability to relevant liver mass, safety, or cell quality [1]. Biotechnological uses face other issues such as price, complexity, duration, high reproducibility, and adaptation to automated high-throughput platforms [2]. Biotechnological applications are broad, including hepatotoxic screening of new molecules or the development of new therapeutic alternatives for severe hepatic diseases.

Conversion of human fibroblasts to induced hepatocyte-like cells (iHEP) can be achieved by overexpression of a set of transcription factors [3–6]. iHEP obtained in different reports display a wide-range expression of genes involved in hepatocyte metabolic functions, reflecting a wide variety of maturation [3, 4]. To achieve high expression of multiple transcription factors, most of the reprogramming protocols use a combination of individual lentivirus each expressing a specific factor. This experimental setting unavoidably results, for each experiment, in a heterologous population of cells expressing multiple transcription factor stoichiometry that severely affects cell homogeneity of the reprogramming experiment [7]. Moreover, the necessity to perform a new round of lentiviral infection in each experiment, limits the use of iHEP in pharmaceutical industry, where adaptation to automated high-throughput screening and Good Manufacturing Practices (GMP) facilities are mandatory [2].

To overcome the hurdles of multiple lentivirus infection in each experiment, we have developed a single polycistronic inducible lentiviral vector able to reprogram synchronously human fibroblasts into iHEP by the simple addition of doxycycline (DOX) to culture media. Manipulated fibroblasts are cultured and expanded in basic media, and only reprogrammed when shifted to hepatic maturation media (HMM) containing DOX. This is a simple and straightforward procedure to generate a homogeneous iHEP population, compatible with FACS or even single-cell cloning to reach a new level of cell homogeneity. When applied to fibroblasts isolated from patients suffering of hepatic metabolic rare diseases, such as Crigler-Najjar or Tyrosinemia, it could be implemented in high-throughput platforms employed for the development of new therapeutic alternatives.

## Methods

### Plasmids and lentivirus generation

**Fig. 1:**
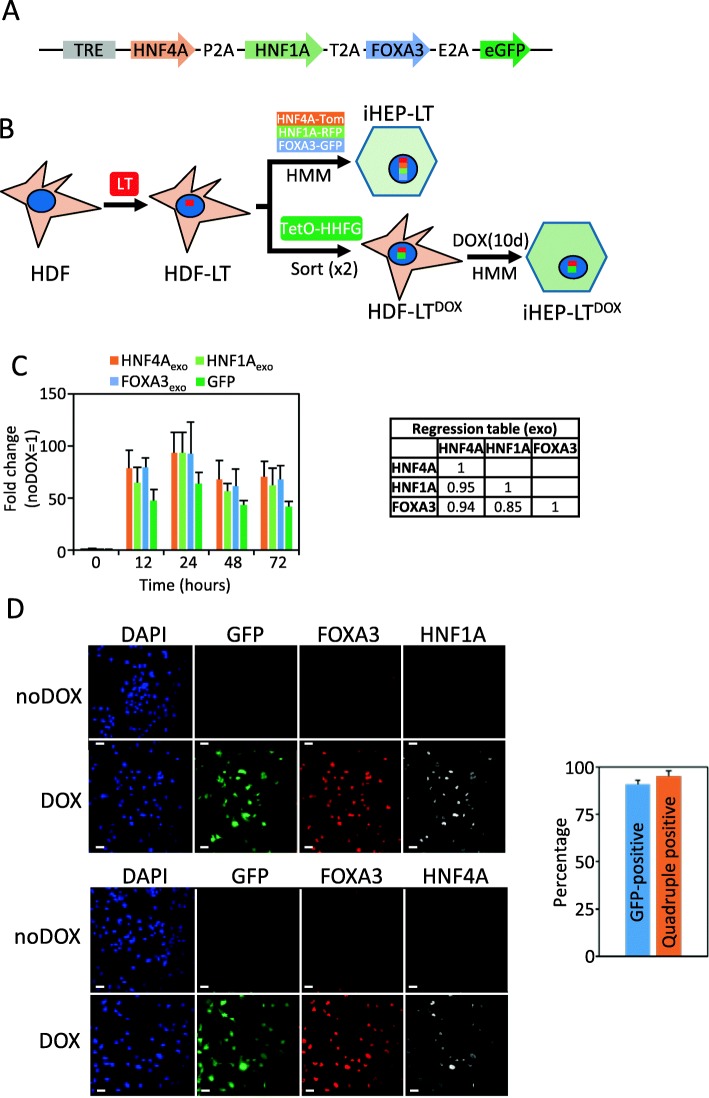
Robust, coordinated induction of HNF4A, HNF1A, FOXA3, and GFP transgenes. **a** Scheme of the DOX-inducible lentiviral vector TetO-HHFG, containing three different 2A peptides. **b** Schematic representation of the reprogramming strategies with and without the inducible reprogramming vector, TetO-HHFG (see “Methods” for details). **c** Early time response of DOX-dependent induction of HNF4A, HNF1A, FOXA3, and GFP mRNA, measured by qRT-PCR in HDF-LT^DOX^ treated with 1000 ng/mL DOX (DOX). Values are referred to untreated HDF-LT^DOX^ (*T* = 0; noDOX). Regression table is on the right. Data shown is represented as mean ± s.d. from two experiments with three biological replicates. **d** Immunofluorescence of HNF4A, HNF1A, FOXA3, and GFP in HDF-LT^DOX^ treated without (noDOX) or with 1000 ng/mL DOX (DOX) for 3 days. Bar equals 50 μm. Quantification of immunofluorescence results is shown on the right

The lentiviral expression vector pRRLsin-SV40 T-mCherry [8] was obtained from addgene (#58993). The lentiviral vector TetO-HHFG (Fig. 1a) was obtained by cloning a synthetic fragment encoding HNF4A-P2A-HNF1A-T2A-FOXA3 (GeneArt®; Thermo Fisher) upstream of the E2A sequence in TetO-FUW-eGFP [9] (Addgene #73083). Reverse tetracycline transactivator expression was achieved using FUW-M2rtTA lentiviral plasmid [10] (Addgene #20342). The lentiviral vector pHIV-EGFP-FOXA3 was obtained by PCR-cloning human FOXA3 cDNA into BamHI-XbaI restriction sites in pHIV-EGFP [11] (Addgene #21373). The lentiviral vector pHIV-dTOM-HNF4A was obtained by PCR cloning human HNF4A cDNA into an EcoRI restriction site in pHIV-dTomato (Addgene #21374). The lentiviral vector pHIV-RFP was obtained by substituting EGFP by synthesized RFP657 sequence [12] in a pHIV-EGFP vector (GeneArt®; Thermo Fisher). The lentiviral vector pHIV-RFP-HNF1A was obtained by cloning human HNF1A cDNA into pHIV-RFP (GeneArt®; Thermo Fisher). All constructs were verified by sequencing. Lentiviruses were generated in 293T cells by cotransfection of pHIV vector with pPAX2 and pMD2.G in a 10:7.5:5 ratio. Lentiviruses were collected and concentrated using a Lenti-X concentrator following manufacturer’s instructions (Clontech).

### Cell culture, sorting, and imaging

Human dermal fibroblasts (HDF) were purchased from ATCC®(CRL-2429). HDF-LT were generated by infecting HDF with lentivirus vector pRRLsin-SV40 T-mCherry [8] (Addgene #58993); after passage 10, the cells were more than 98% mCherry-positive. HDF-LT were infected with 1:1 mixture of reprogramming lentivirus generated with TetO-HHFG and FUW-M2rtTA. The fraction of infected cells was ≈ 60% as determined in a separate well by GFP positivity after a 24-h pulse of 1 μg/mL of doxycycline (DOX). Cells were expanded and those constitutively expressing GFP above background (≈ 18%) eliminated by cell sorting using a BDFACSAria III™ cell sorter (Additional file 1: Figure S1A). Selected cells were then expanded and GFP-positive sorted after a 24-h pulse of 1 μg/mL DOX and seeded without DOX to obtain HDF-LT^DOX^ (Additional file 1: Figure S1B). HDF-LT^DOX^ were maintained in DMEM. All media was purchased from Thermo Fisher. Growth factors were purchased to Peprotech. Cells were maintained at 37 °C with 5% CO_2_ and were regularly examined with an Olympus CKX41 microscope.

Cell proliferation was assessed as previously described [13].

### iHEP reprogramming

Reprogramming of HDF-LT was performed as described [4]. Briefly, cells were infected with lentiviral vectors independently expressing HNF4A, HNF1A, and FOXA3 (Fig. 1b). After 2 days cultured in HFM media, cell media was shift to HMM for an additional 10 days to obtain iHEP-LT. iHEP-LT^DOX^ were obtained by incubating HDF-LT^DOX^ in HMM media containing 250 ng/mL DOX for 10 days (Fig. 1b).

### qRT-PCR, immunofluorescence, PAS staining, and albumin secretion

qRT-PCR, immunofluorescence, and periodic acid-Schiff (PAS) staining were performed as previously described [14]. A control liver RNA sample was obtained by combining human liver total RNA (Thermo Fisher) from three donors. Primer sequences are shown in Additional file 2: Table S1. Exogenous expression of HNF4A, HNF1A, and FOXA3 (*exo* primers) was determined by designing the forward primers within the lentiviral vector sequence and the reverse primers close to the 5′-end of the corresponding coding sequences. *Tot* primers were designed within the coding sequence of the gene. Fluorescence images were taken in Olympus FV1000 confocal mounted on an IX81 inverted microscope. Quantification of immunofluorescence results was performed using Cellprofiler software [15].

To determine the presence of human albumin in cell media and mice sera, we used a human Albumin ELISA Quantitation Set (Bethyl Laboratory) according to the manufacturer’s instructions. Reference value (primary cultured human hepatocytes) was extracted and adapted from previously published data from our group [16].

### Glutamine and glutamate determination in cell media by liquid chromatography high-resolution mass spectrometry (LC-HRMS) analysis

Chromatographic analysis was performed on an Agilent 1290 Infinity II (Agilent Technologies, Santa Clara, CA, USA) HPLC system equipped with a quaternary pump, vacuum degasser, and autosampler with a temperature controller. Chromatographic separation of metabolites was achieved on a 150 mm × 2.1 mm, 4 μm particle size Synergi-Hydro C18 column (Phenomenex Inc., Torrance, CA, USA) with the following separation conditions: solvent A, water/FA (99.8:0.2); solvent B, ACN; separation gradient, initially 1% B, held for 2 min and then linear 1–80% B in 8 min, washing with 98% B for 2 min and column equilibration with 1% B for 7 min; flow rate, 0.25 mL/min; injection volume range, 0.2–4.5 μl. Autosampler and column temperatures were set at 6 °C and 23 °C, respectively.

Mass spectrometry analysis was carried out by an Agilent 6550 Q-ToF (Agilent Technologies, Santa Clara, CA, USA) detector equipped with an electrospray ionization (ESI) source with Jet Stream Technology. Column flow was conducted into the mass analyzer in the time range of 0.7–12 min diverting the rest of run time to waste. MS conditions of analysis were as follows: gas temp, 130 °C; drying gas, 14 L/min; nebulizer, 30 psig; sheath gas, 10 L/min; capillary voltage, 3500 V and 3000 V for positive and negative ionization modes, respectively; fragmentor, 380 V; octapole 1 RF, 400 V; isolation width, narrow (1.3 m/z); nozzle voltage, 500 V funnel exit DC, funnel RF HP, and funnel exit RF LP, 50, 150, and 60 V, respectively; lock masses, 119.0363/980.0164; considered m/z range, 40–750; data acquisition, centroid mode.

Before sample analysis, the MS device was tuned and calibrated in low mass range and high-resolution mode (4 GHz). Considered mass tolerance for full MS and MS/MS analyses for data processing was 10 ppm. Absolute quantification of glutamic acid and glutamine was carried out through their respective relative response factors using D5-glutamic and D4-succinic acids as IS, respectively.

To assess glutamine and glutamate concentration in cell media, 24-h HMM media from iHEP-LT^DOX^ was collected and immediately frozen in liquid N_2_, and kept at − 80 °C until analysis. Before analysis, samples were diluted 1/100 with water containing D4-succinic acid, D5-glutamic acid, and D5-phenylalanine as internal standards (IS; final concentration 2 ppm) and filtered through a modified PES 3K molecular exclusion filter (VWR; Radnor, PA, USA). Glutamine uptake was determined by subtracting glutamine concentration in media from control plates (without cells) and 24-h incubation media. Glutamate secretion was determined by subtracting glutamate concentration in 24-h incubation media and media from control plates (without cells).

### In vivo transplantation

Transplantation of iHEP in male CB17/Icr-Prkdc scid/Crl mice was done as previously described [17]. Animals were acquired from Charles River Laboratories and housed at the animal facilities of the Instituto de Investigación Sanitaria La Fe. Experimental protocols were approved by the Institutional Animal Ethics Committee of the Instituto de Investigación Sanitaria La Fe and Generalitat Valenciana (reference number IP.RBM.#6A-3-2015). Briefly, 3 h after the injection of 400 mg/kg of acetaminophen (APAP), mice were anesthetized with a sevoflurane/O_2_ mixture and the lower pole of the spleen was exposed. Animals received an intrasplenic injection of 10^6^ HDF-LT^DOX^, iHEP-LT^DOX^, or iHEP-LT in 200 μl of phosphate-buffered saline within seconds. Mice were kept with 2 mg/mL DOX in drinking water. Blood was collected at 7 and 30 days, and serum aliquots were protected from light and stored at − 80 °C until analysis. Thirty days after infusion, mice were sacrificed under anesthesia (sevoflurane/O_2_ mixture).

## Results

### DOX-inducible coordinated expression of hepatic transcription factors

A tetracycline-inducible vector (TetO-HHFG) was constructed based on the TetO-FUW-eGFP plasmid where gene expression is controlled from seven copies of the tetracycline operator (7xTetO). To achieve coordinated expression of the inserted genes, HNF4A, HNF1A, and FOXA3 were cloned upstream of GFP cDNA and separated by P2A, T2A, and E2A sequences [18] (Fig. 1a). To validate our DOX-inducible lentiviral setting, normal human dermal fibroblasts (HDF) were infected with TetO-HHFG, and the reverse tetracycline-controlled transactivator (rtTA) lentiviral vectors. Cells were cultured in HMM media as described in the “Methods” section and the expression of the exogenous genes and albumin evaluated by immunocytochemistry. GFP-positive cells co-expressed HNF4A/ALB or FOXA3/HNF1 (Additional file 1: Figure S2). Due to the big size of the insert, infectivity was low and variable (5–27%). This drawback could be effectively circumvented by GFP-sorting of the infected fibroblasts.

HDF were first immortalized by expression of the large-T antigen (HDF-LT) to avoid cell senescence and to permit expansion and stocking of the infected cells after cell sorting. HDF-LT were co-infected with TetO-HHFG and the reverse tetracycline-controlled transactivator (rtTA) lentiviral vectors. Cells were expanded several folds in the absence of DOX and GFP-sorted twice as described in “Methods” (Fig. 1b and Additional file 1: Figure S1). Thereafter, cells were cultured and expanded in the absence of DOX to obtain HDF-LT^DOX^ line.

To test whether all three factors were expressed upon DOX addition, HDF-LT^DOX^ were treated with DOX (1 μg/mL) and RNA was isolated at 12, 24, 48, and 72 h. Using primers specific for the lentiviral-expressed inserts (*exo* primers; Additional file 2: Table S1), a fast, robust, and coordinated induction was observed in DOX-treated cells (Fig. 1c). To test their relative expression compared to human liver, primers that do not discriminate between exogenous and endogenous transcripts were used (*tot* primers; Additional file 2: Table S1) and the relative expression were determined for HNF4A (≈ 20-fold), HNF1A (≈ 30-fold), and FOXA3 (≈ 40-fold). Immunofluorescence analysis evidenced little or no expression in untreated cells, while robust protein induction was observed in nuclei of cells treated with DOX (Fig. 1d).

### Generation of a DOX-reprogrammable immortal human fibroblast cell line

**Fig. 2:**
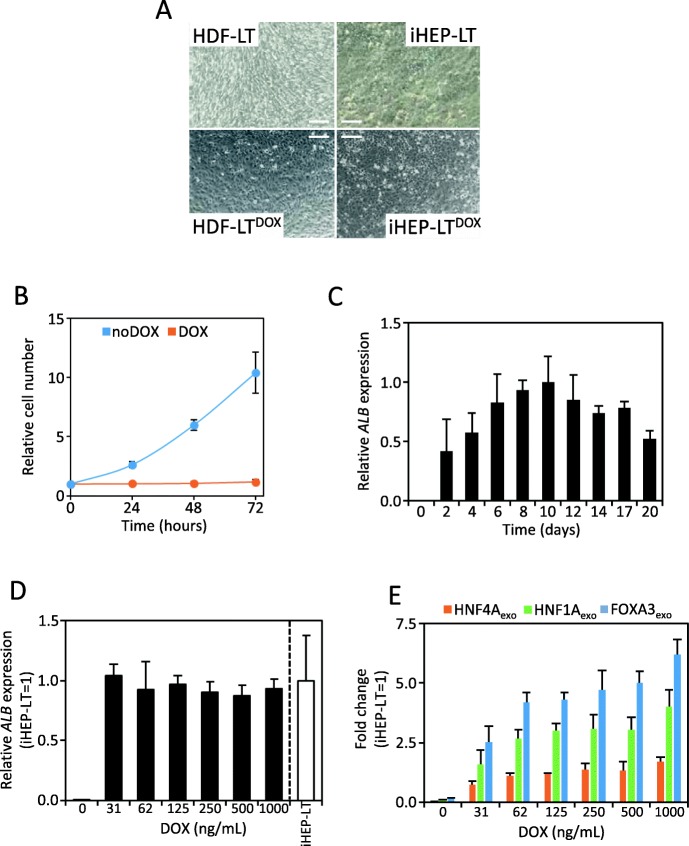
Generation of human iHEP using a single polycistronic lentivirus. **a** Morphological changes after HDF and HDF-LT reprogramming to iHEP (see Fig. 1b for details). **b** DOX treatment halted cell proliferation. HDF-LT^DOX^ were treated with 1000 ng/mL DOX and cells were counted by cell cytometry at different time points. **c** HDF-LT^DOX^ were treated with 1000 ng/mL DOX and RNA isolated at multiple time points. *ALB* mRNA expression was quantified by qRT-PCR and expressed relative to the expression at 10 days. **d**, **e** HDF-LT^DOX^ were treated with different concentrations of DOX and RNA isolated after 10 days. *ALB* and exogenous transgene expression was quantified by qRT-PCR and expressed relative to iHEP-LT levels (see Fig. 1b for details)

To address whether DOX addition to cell media was able to directly convert HDF-LT^DOX^ into iHEP (iHEP-LT^DOX^), cells were cultured in the presence of 1 μg/mL DOX. Basal expression of HNF4A, HNF1A, and FOXA3 from the tetracycline promoter induced a minimal change in cell morphology in HDF-LT (Fig. 2a). DOX caused a sharp decrease in HDF-LT^DOX^ proliferation already detectable after 48 h (Fig. 2b), a characteristic of iHEP [4]. Time-course analysis revealed that *ALB* mRNA expression was already detected as early as 2 days after DOX addition reaching a maximum level after 10 days (Fig. 2c). Extended cell culture did not increase albumin expression, rather it was reduced by more than 25% beyond day 12. In fact, keeping the reprogramming conditions beyond 14 days resulted in poor-quality cultures. Next, we estimated the concentration of DOX required to induce albumin after 10 days of treatment. We examined a dose range of 30–1000 ng/mL. No significant difference in albumin expression was found among the different doses (Fig. 2d); as low as 30 ng/mL induces a robust albumin expression comparable to the independent lentiviral expression of the transcription factors (iHEP-LT). We selected the highest DOX concentration without morphological evidences of cell culture quality decline, i.e., 250 ng/mL. Nevertheless, induction of HNF4A, HNF1A, and FOXA3 was still dose-dependent (Fig. 2e).

**Fig. 3:**
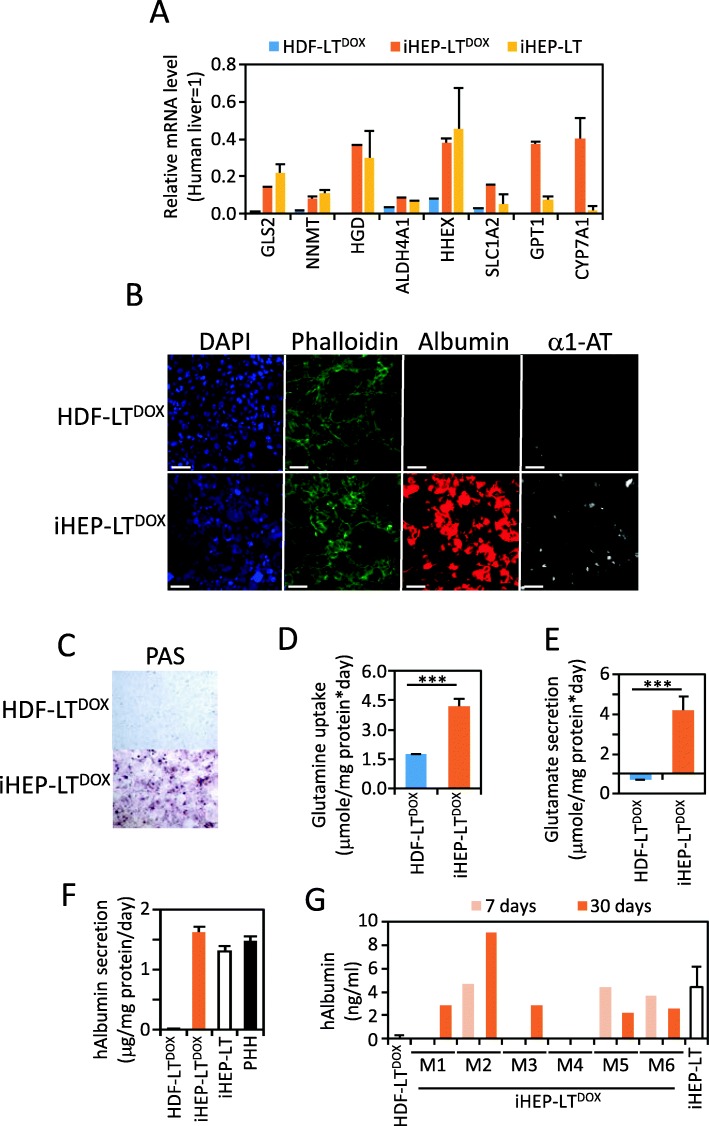
Characterization of iHEP^DOX^. HDF-LT^DOX^ were reprogrammed to iHEP-LT^DOX^ by incubation in HMM media containing 250 ng/mL DOX for 10 days. iHEP-LT are included as reference (see Fig. 1b for details). **a** mRNA level of multiple hepatic genes was quantified by qRT-PCR and expressed relative to human liver. **b** Representative fluorescence images of cells immunostained with antibodies against human albumin and a1-antitrypsin. Nuclei were stained with DAPI. Bar equals 40 μm. **c** PAS staining. **d** Glutamine uptake from cell media. **e** Glutamate secretion to cell media. **f** Human albumin contained in 24-h cell media was quantified by ELISA. PHH: cultured primary human hepatocytes (data extracted from [16]). **g** Human albumin present in sera from cell transplanted SCID mice quantified by ELISA at 7 and 30 days. M: mouse number

Reprogrammed iHEP-LT^DOX^ expressed multiple hepatic genes at a level similar to iHEP-LT and in the range of liver mRNA levels (Fig. 3a). De novo expression of liver-specific phosphate-activated glutaminase (GLS2), homogentisate 1,2-dioxygenase (HGD) involved in tyrosine or phenylalanine catabolism in the liver, and cytochrome P450 CYP7A1 responsible for the rate-limiting step of bile acid synthesis from cholesterol or glutamic-pyruvic transaminase (GPT1) are remarkable. Reprogrammed cells expressed albumin and a1-antytrypsin (Fig. 3b), accumulated glycogen (Fig. 3c), performed glutamine/glutamate conversion and secretion (Fig. 3d, e), and secreted albumin to the level of primary cultured human hepatocytes (Fig. 3f). When 2-day treated HDF-LT^DOX^ were transplanted in immunosuppressed mice, human albumin was clearly detected in sera (Fig. 3g). hAlbumin levels changed over time resulting in lower expression in two mice and higher expression in three mice. Nevertheless, levels remained fairly stable suggesting that cells are not eliminated over time. One of the animals (M4) was not significantly colonized by iHEP-LT^DOX^.

### High expression of exogenous factors is required to maintain full iHEP identity

**Fig. 4:**
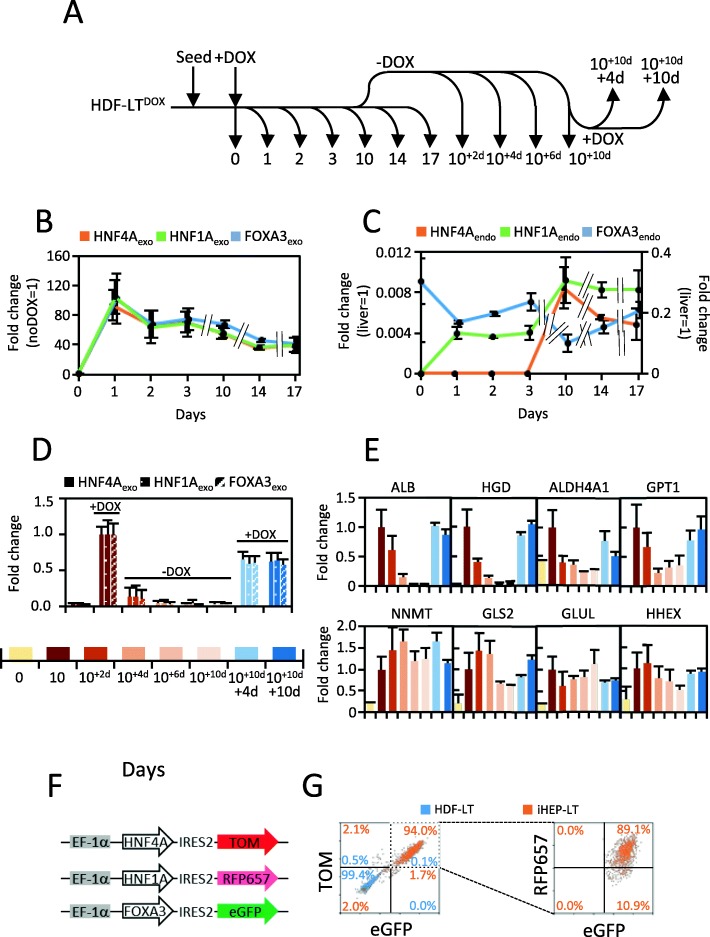
Phenotype reversal of iHEP-LT^DOX^. **a** Schematic representation of time-course collection of reprogramming intermediates. Reprogramming of HDF-LT^DOX^ was initiated at day 0 by addition of DOX 250 ng/mL. The collection point labeled 10^+d^ represents cells reprogrammed for 10 days in DOX followed by specified days (in superscript) without DOX. **b**, **c** Expression of exogenous and endogenous reprogramming factors across a 0–17-day timeline. **d**, **e** Expression of exogenous transgenes and selected hepatic mRNA during the timeline described in **a**. mRNA levels were quantified by qRT-PCR and expressed relative to the expression at 10 days. **f** Scheme of the bicistronic lentiviral vectors independently expressing HNF4A, HNF1A, and FOXA3 together with mutually compatible fluorescent proteins. **g** HDF-LT cells were co-infected with HNF4A, HNF1A, and FOXA3 lentiviral vectors and cultured in HMM media. Expression of fluorescent proteins was assessed by flow cytometry after 30 days

We next determined the expression of exogenous factors across cell reprogramming timeline (Fig. 4a). A gradual decrease of exogenous HNF4A, HNF1A, and FOXA3 was observed along the reprogramming process, reaching a 50% decrease after 17 days (Fig. 4b). Noteworthy, the expression of endogenous FOXA3 and HNF4A were below 1% of that of human liver at all time points assayed, and, only endogenous HNF1A reached the range of human liver (Fig. 4c). These results suggest that phenotype conversion and maintenance relied on expression of exogenous transgenes rather than endogenous taking over. Beyond the complete silencing of exogenous factors in reprogrammed cells, DOX-inducible systems offer an unbiased tool to definitively assess cellular phenotype dependence on expressed transgenes [19]. Removal of DOX after a 10-day reprogramming downregulated exogenous transgene expression, reaching levels comparable to untreated cells after 6–10 days (Fig. 4d). In parallel, we observed a sharp downregulation of ALB, HGD, ALDH4A1, and GPT1 to levels of untreated cells (Fig. 4e). Interestingly, other hepatic markers such as NNMT, GLS2, GLUL, and HHEX maintained elevated mRNA levels in the absence of DOX. This variable dependence upon exogenous factor expression is suggestive of partial reprogramming of iHEP-LT^DOX^. We also tested the capacity of the inducible system and cells to respond to DOX re-addition. Reprogramming factors were re-expressed to 50% of the original level, suggesting a partial refractoriness of the inducible system after a first round of DOX. Nevertheless, hepatic-specific gene induction remained unchanged (Fig. 4d, e).

We next hypothesized that uncomplete reprogramming might be the consequence of the difference between polycistronic DOX-inducible coordinated expression system and independent expression in non-inducible vectors. To explore this hypothesis, we generated iHEP-LT using the three lentiviral vectors coexpressing each transcription factor and mutually compatible fluorescent proteins from a non-inducible bicistronic cassette (Fig. 4f). Maintained co-expression in 84% of the cells was evident after 30 days by flow cytometry and qRT-PCR (Fig. 4g and data not shown).

## Discussion

Our work proves that DOX-dependent coordinated expression of three transcription factors separated by 2A sequences can convert fibroblasts to induced hepatocyte-like cells (iHEP-LT^DOX^). These cells perform hepatic-specific functions similar to fibroblasts reprogrammed by coinfection of three lentiviral vector constitutively expressing the same reprogramming factors individually (iHEP-LT). However, morphological and cell division differences could be observed. Slight epithelization of HDF-LT^DOX^ fibroblasts cultured in the absence of DOX is observed at phase contrast microscopy when compared with HDF-LT, probably caused by leaky expression of the reprogramming factors from the TRE promoter. In fact, HNF4A expression confers epithelial phenotype on fibroblasts cells [20]. Nevertheless, phalloidin staining shows a more cortical organization of actin filaments characteristic of epithelial cells in iHEP-LT^DOX^ compared to actin stress fibers found in HDF-LT^DOX^ (Fig. 3b). iHEP-LT proliferate in culture, while HDF-LT^DOX^ halt proliferation as early as 24 h after DOX addition. In fact, we routinely maintain and amplify HDF-LT^DOX^ by subculturing under usual fibroblast culture conditions and only add DOX and specific HMM media when reprogramming is needed. Such difference might be due to higher expression of transcription factors in iHEP-LT^DOX^ or the different stoichiometry between HNF4A, HNF1A, and FOXA3 expression in both cell types. While polycistronic 2A system supports almost equimolar mRNA expression [21] (Fig. 1c), independent expression with our lentiviral vectors always result in higher FOXA3 expression (Fig. 2e).

Another important conclusion of our study is that reprogramming of fibroblasts to iHEP by ectopic expression of HNF4A, HNF1A, and FOXA3 is not complete. While expression of endogenous HNF1A in iHEP-LT is within the range of human liver, expression of endogenous HNF4A and FOXA3 is low. These results are in agreement with the data presented in the original report [4] and reproduced in iHEP-LT generated in our laboratory (data not shown). Secondly, DOX withdrawal results in downregulation of conversion factors and dramatic silencing of critical hepatic genes such as *ALB* or *HGD*. Nevertheless, other hepatic genes remain stably expressed. Such variability might be the consequence of the hierarchical binding of HNF4A, HNF1A, and FOXA3 to the gene-specific promoters. For example, FOXA binding is critical for ALB mRNA transcription [22, 23]. It could also be related to the resistance to epigenetic rewiring of highly hepatocyte-specific genes such as ALB and HGD.

An important and disregarded aspect of direct cell conversion is how close the expression of reprogramming factors is to the endogenous expression in the target cell. We found that conversion factor mRNAs from the lentiviral vector are expressed 15–40 fold higher than those endogenously expressed in human hepatocytes. This result is comparable to DOX-inducible polycistronic reprogramming of fibroblasts into induced pluripotent stem cells (iPSC) [18], where 20-fold expression of these factors in hESC is obtained in mouse fibroblasts 48 h after transduction. However, while DOX-induced iPSC reprogramming does not require maintained DOX expression beyond day 13, iHEP lose their phenotype after DOX withdrawal.

## Conclusions

In summary, we report the generation of a fibroblastic cell line, HDF-LT^DOX^, immortalized and fully expandable that converts to a hepatocyte-like phenotype upon DOX addition to cell media. Derivation of similar cell lines from fibroblasts obtained from patients suffering of liver-related congenital rare diseases could be a valuable tool for screening new therapeutic approaches.

## Supplementary information

**Additional file 1:** **Figure S1.** Cell sorting strategy using BDFACSAriaIII™ cell sorter. **Figure S2.** Validation of the polycistroniclentiviral vector in HDF.

**Additional file 2:** **Table S1.** Primers used for qRT-PCR.

## Data Availability

The datasets used and/or analyzed during the current study are available from the corresponding author on reasonable request.

## Abbreviations

APAP: Acetaminophen
DMEM: Dulbecco’s modified Eagle’s media
DOX: Doxycycline
ESI: Electrospray ionization
FACS: Fluorescence-activated cell sorting
GMP: Good Manufacturing Practices
GFP: Green fluorescent protein
hESC: Human embryonic stem cell
HFM: Human fibroblast medium
HMM: Hepatic maturation media
HDF: Human dermal fibroblasts
iHEP: Induced hepatocyte-like cells
iPSC: Induced pluripotent stem cell
LT: Large-T antigen
MS: Mass spectrometry
PAS: Periodic acid-Schiff
TetO: Tet operator
rtTA: Tetracycline-controlled transactivator
