# Supplementary material for: Direct conversion of human fibroblast to hepatocytes using a single inducible polycistronic vector
Source: Stem Cell Res Ther. 2019 Nov 4;10:317. doi: 10.1186/s13287-019-1416-5 (PMC6829923; doi:10.1186/s13287-019-1416-5)
Supplement: Supplementary file 1 — Additional file 1: Figure S1. Cell sorting strategy using BDFACSAriaIII™ cell sorter. Figure S2. Validation of the polycistroniclentiviral vector in HDF. [file 13287_2019_1416_MOESM1_ESM.pdf]

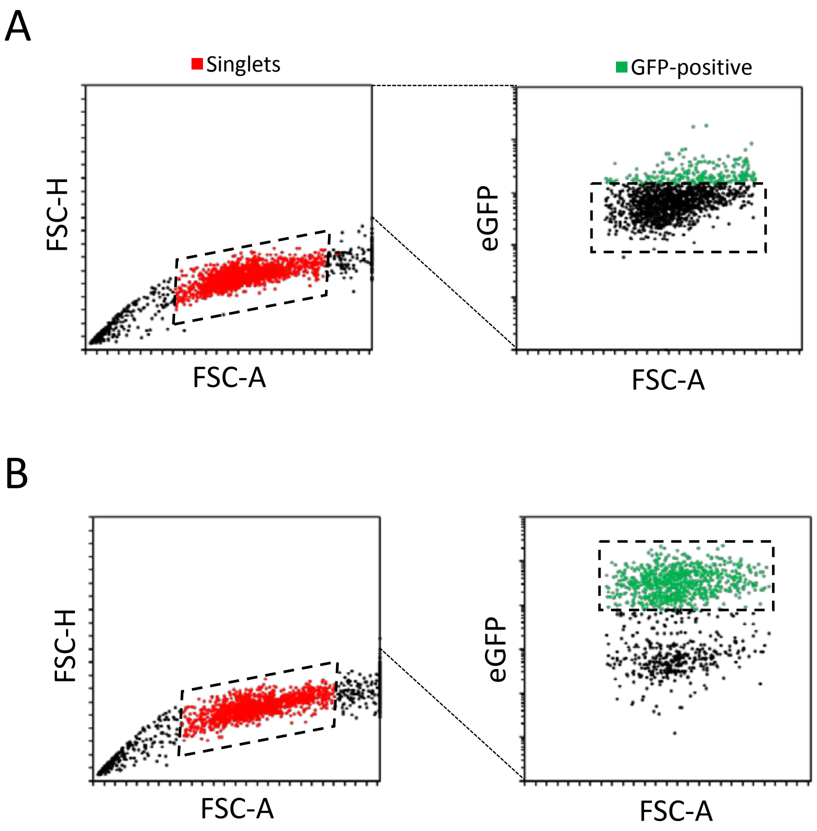

Figure S1. Cell sorting strategy using BDFACSAria III™ cell sorter. HDF-LT were infected with 1:1 mixture of reprogramming lentivirus generated with TetO-FUW-HHFG and FUW-M2rtTA. **(A)** Single cells constitutively expressing GFP above background were sorted out and discarded. The rest were expanded in culture. **(B)** Cells expanded from (A) were treated with a 24-hr pulse of 1µg/mL DOX and single cells expressing GFP sorted for expansion in culture without DOX to obtain HDF-LT^{DOX}. Boxes represent cells selected for further culture.

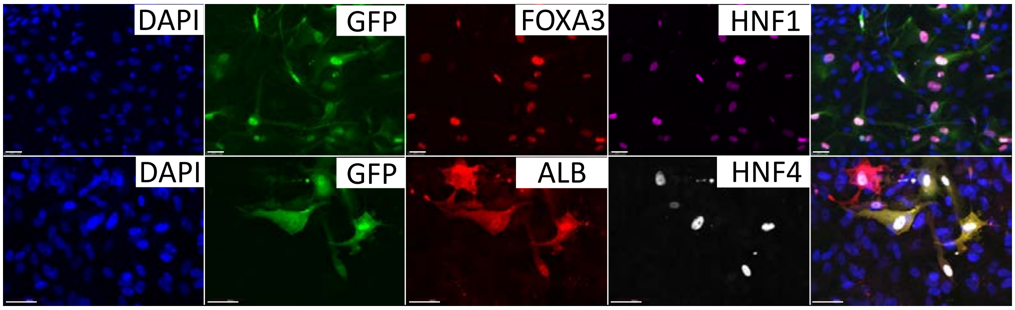

Figure S2. Validation of the polycistronic lentiviral vector in HDF. HDF cells were infected with 1:1 mixture of TetO-FUW-HHFG and FUW-M2rtTA and reprogrammed using HMM media for 10 days. Representative fluorescence images of cells immunostained with antibodies against GFP, human albumin, HNF4, HNF1 and FOXA3 are shown. Nuclei were stained with DAPI. Bar equals 50 µm.
