# Supplementary material for: Direct conversion of human fibroblast to hepatocytes using a single inducible polycistronic vector
Source: Stem Cell Res Ther. 2019 Nov 4;10:317. doi: 10.1186/s13287-019-1416-5 (PMC6829923; doi:10.1186/s13287-019-1416-5)
Supplement: Supplementary file 2 — Additional file 2: Table S1. Primers used for qRT-PCR. [file 13287_2019_1416_MOESM2_ESM.docx]

**Table S1. Primers used for qRT-PCR**

| **Transcript** | **Forward (5'-3')** | **Reverse (5'-3')** | **Size (bp)** |
| --- | --- | --- | --- |
| HNF4Aexo | ATCCACGCTGTTTTGACCTC | CTCAAATTCCAGGGTGGTGT | 158 |
| HNF1Aexo | CGCCACGAACTTCTCTCTGT | GGATCAGTGCCTCTTTGCTC | 140 |
| FOXA3exo | AGGGCAGGGGAAGTCTTCTA | AGCTTAGAGGATTCAGGGTCA | 195 |
| HNF4Aendo | CAAGTGCCCCTGAAATCCCT | CGGCACTCCATGGGACATTC | 165 |
| HNF1Aendo | AGCGTCATCGAGACCTTCAT | GATGCATCAGAGCAGAGTGGG | 183 |
| FOXA3endo | CTAGCAGGGGTTGGGAACAT | ATCAACACCATGCCCACTGA | 164 |
| HNF4Atot | CATGGACATGGCCGACTACA | CTCGAGGCACCGTAGTGTTT | 201 |
| HNF1Atot | CACCAAGCAGGTCTTCACCTC | TCTCGATGACGCTGTGGTTG | 245 |
| FOXA3tot | AGTGGAGCTACTACCCGGAG | AGCTTAGAGGATTCAGGGTCA | 101 |
| ALB | TAAGGAGACCTGCTTTGCCG | AGACAGGGTGTTGGCTTTACA | 183 |
| GLS2 | CGAAGGTTTGCCTTGTCAGC | AGAGTGTGTAGGAGTCCTGGT | 238 |
| NNMT | GACTACTCAGACCAGAACCTGC | GTCACATCACACTTCAGCACCT | 179 |
| HGD | GGACCTGATGCTGACTGCTT | AAGTGGCTCTTGAGTGGCTC | 188 |
| ALDH4A1 | CTGGACGGGGTTGTGCATT | CCCTGCGTGAAGGCTAAGAC | 199 |
| HHEX | CGGACGGTGAACGACTACA | TTTGACCTGTCTCTCGCTGA | 234 |
| SLC1A2 | GCTGCACACAACTCTGTCAT | GCAGTCGGCTGACTTTCCAT | 72 |
| GPT1 | CTCTCTTGCCTGGAGTTCCCTC | CTCGAGGCCATGACTCTACCC | 148 |
| CYP7A1 | GCATGCTGTTGTCTATGGCTT | AACTCAAGAGGATTGGCACCA | 124 |
| PBGD | CGGAAGAAAACAGCCCAAAGA | TGAAGCCAGGAGGAAGCACAGT | 272 |
| GAPDH | ATGCTGGCGCTGAGTACGTC | TGACCTTGGCCAGGGGTGCT | 224 |
